# Conversation for change: engaging older adults as partners in research on gerotechnology

**DOI:** 10.1186/s40900-024-00557-3

**Published:** 2024-02-20

**Authors:** Jessica Bytautas, Alisa Grigorovich, Judith Carson, Janet Fowler, Ian Goldman, Bessie Harris, Anne Kerr, Ashley-Ann Marcotte, Kieran O’Doherty, Amanda Jenkins, Susan Kirkland, Pia Kontos

**Affiliations:** 1 Dalla Lana School of Public Health, University of Toronto, Toronto, Canada; 2 KITE Research Institute, Toronto Rehabilitation Institute – University Health Network, Toronto, Canada; 3 Recreation and Leisure Studies, Brock University, St. Catharines, Canada; 4 Community Partner, Guelph, Canada; 5 Community Partner, Red Deer, Canada; 6 Community Partner, Toronto, Canada; 7 Community Partner, Halifax, Canada; 8 Department of Community Health and Epidemiology, Dalhousie University, Halifax, Canada; 9 Department of Psychology, University of Guelph, Guelph, Canada

**Keywords:** Older adults, Gerotechnology, Participatory action research, Aging, Stigma

## Abstract

There is increasing research and public policy investment in the development of technologies to support healthy aging and age-friendly services in Canada. Yet adoption and use of technologies by older adults is limited and rates of abandonment remain high. In response to this, there is growing interest within the field of gerotechnology in fostering greater participation of older adults in research and design. The nature of participation ranges from passive information gathering to more active involvement in research activities, such as those informed by participatory design or participatory action research (PAR). However, participatory approaches are rare with identified barriers including ageism and ableism. This stigma contributes to the limited involvement of older adults in gerotechnology research and design, which in turn reinforces negative stereotypes, such as lack of ability and interest in technology. While the full involvement of older adults in gerotechnology remains rare, the Older Adults’ Active Involvement in Ageing & Technology Research and Development (OA-INVOLVE) project aims to develop models of best practice for engaging older adults in these research projects. In this comment paper, we employ an unconventional, conversational-style format between academic researchers and older adult research contributors to provide new perspectives, understandings, and insights into: (i) motivations to engage in participatory research; (ii) understandings of roles and expectations as research contributors; (iii) challenges encountered in contributing to gerotechnology research; (iv) perceived benefits of participation; and (v) advice for academic researchers.

## Abstract (plain language summary)

More investments are being made to develop technologies that support healthy aging and age-friendly services in Canada. However, not many older adults use these technologies and those who do tend to stop using them after some time. Gerotechnology is a field of study that combines an interest in gerontology and technology. Within gerotechnology, researchers are learning more about how to encourage older adults to participate in research and the design of new technologies. There are different ways that older adults participate in gerotechnology research, with some approaches being more passive than others. In participatory design and participatory action research projects older adults are encouraged to engage more actively as co-researchers. However, researchers have found that there are some limitations to engaging older adults actively in research, including ageism and ableism, meaning that older adults are perceived to be capable of contributing based on their age and cognitive or physical abilities. These stereotypes have limited how often and how much older adults actually contribute to technology research and design. The Older Adults’ Active Involvement in Aging & Technology Research and Development (OA-INVOLVE) project aims to address these gaps. In this comment paper, we present a conversation between academic and older adult researchers who have contributed to OA-INVOLVE. The goal of this conversation is to explore together: (i) motivations to engage in participatory research; (ii) understandings of roles and expectations as research contributors; (iii) challenges encountered in contributing to gerotechnology research; (iv) perceived benefits of participation; and (v) advice for academic researchers.

## Background

There is increasing research and public policy interest in the development of technologies to support healthy aging and age-friendly services in Canada [1, 2]. Yet adoption and use of technologies by older adults is low and rates of their abandonment remain high [3, 4]. Limited uptake has largely been attributed to the fact that gerotechnologies tend to reflect the needs and preferences of designers and researchers, rather than those of older adults [1]. This has prompted calls within gerotechnology for greater participation of older adults in research and design of services and products using various approaches such as participatory action research, co-design, user-centred design, and participatory design (PD) [3, 5]. The nature of older adults’ participation in research studies that use these participatory approaches ranges greatly from passive information gathering (e.g. needs assessment studies) to more active involvement in design decisions and activities. The latter is key to ensuring that new technologies reflect older adults’ unique needs, capabilities, limitations, and preferences.

PD has been described as “designing WITH users” [6] and democratizing the design of technologies [5]. PD is often described as being consistent with or derived from participatory action research (PAR) [7]. PAR is a specific subset of action research that is emancipatory and focuses on effecting structural change by shifting the balance of power from academic and scientific stakeholders, to communities who may experience structural oppression [8]. PAR is a critically oriented research approach focused on social justice in which experiential stakeholders participate as equal partners (co-researchers) with decision-making power in all aspects and phases of the research [9] rather than as research subjects or participants [10]. Researchers must intentionally make participation accessible by removing barriers to participation and by engaging communities in discussion regarding how they would like to participate [11, 12]. Through their active participation, community stakeholders become empowered to effect social change that can range from consciousness raising to changes in practices and policies. Such participation not only yields new skills and competencies but increases one’s sense of control, involvement in decision making, and critical awareness.

Despite the increased interest in PAR and its noted benefits, this approach is rare [5]. Multiple barriers to participatory involvement of older adults in gerotechnology research have been identified, with the majority of the research focusing on barriers stemming from ageism and ableism. More specifically, researchers have identified as barriers the assumptions that older adults are “technology inept and digitally illiterate” [13] and that their lower use of technologies is due to age-related functional limitations (e.g., vision, hearing, and touch) or their negative attitudes towards such limitations [14]. This stigma contributes to the limited involvement of older adults in gerotechnology, which in turn reinforces the negative stereotype of older adults as being incapable or uninterested in technology and its development.

While the full involvement of older adults in gerotechnology research remains rare (i.e., from project conception to dissemination), the Older Adults’ Active Involvement in Ageing & Technology Research and Development (OA-INVOLVE) project [15] is a noteworthy exception. It actively supports older adults to participate as co-researchers in their own projects by having a distributed governance model that includes an Older Adult Research Partner Group (OARPG) as well as by providing opportunities for older adult researchers to build their research and digital literacy skills in conducting data collection and analysis, as well as knowledge translation [16]. Some examples of this include older adults interviewing researchers about the degree of engagement of older adults in their projects, training older adults to participate in qualitative data analysis, and older adults and researchers co-presenting at conferences. Additionally, OA-INVOLVE and the OARPG have developed documents, reports, and guidelines to help other researchers and older adults effectively engage with each other in research. [9]

In this article, we deliberately chose to employ a novel conversational style for which there is a precedent in gerontological research [17, 18]. This style best supports our interest in engaging older adults about their involvement in the development of technology research and design as research partners rather than as study participants. Further, we felt that a conversational approach would offer an alternative and more engaging format to increase the accessibility of the ideas shared and to facilitate broader engagement with them. Academic and older adult researchers from across Canada and who have participated in OA-INVOLVE met virtually over several months to discuss their motivations, understandings, challenges, benefits, and advice for others who may wish to engage in such collaborations. These discussions were edited for clarity and brevity to form this conversation. Overall, our aim is to prompt others within the gerotechnology field to bring about greater opportunities for all older adults to equally engage and learn, and to have their values and experiences incorporated in technology design and development processes.

Interlocutors are identified in parentheses as either academic researchers (AR) or older adult researchers (OA). The ages of interlocutors ranged from 71 to 85 years for the older adults and 32 to 67 years for the academic researchers; specific ages are indicated in parentheses after each interlocutor’s name. In terms of education level, all interlocutors have a postsecondary certificate, diploma or degree. The racial/ethnic group with which all interlocutors identify with is White. All but two interlocutors identified as women.

## Motivations to engage in research

- Alisa (AR 41): I came to the idea of co-design in aging and technology in a roundabout way. I was already interested in participatory forms of health services research with older adults way before I ever knew anything about technology. I understand very well that researchers are experts in methods and theory, but without engaging older adults and other publics, how can we know whether our research questions and outputs are valuable or useful?
- Kieran (AR 49): Science certainly has the capacity to bring great improvements to our lives, and this is evidently so in the case of technologies designed to assist us into old age. But who is involved in the science and design decisions that go into developing these technologies? When I joined OA-INVOLVE, I saw an opportunity to learn new and meaningful ways to build partnerships between scientists and broader publics.
- Alisa (AR 41): Imagine my surprise when I realized that not only is participatory design very rare in aging and technology research, most research and development of technologies is not participatory at all!
- Pia (AR 54): Engaging older adults as co-researchers is definitely still pretty novel in the field of technology. I’m drawn to this type of work because of a deep concern about stigma associated with aging, which creates discriminatory and exclusionary practices, including in research. Researchers don’t live in a bubble, so they are very much wrapped up in societal assumptions about aging, which inform the ways that they include (or not) older adults in research.
- Anne (OA 79): It was important for me to get involved with research in order to counteract stereotypes and prejudices related to aging, particularly assumptions that as an older adult I have nothing to contribute. Out in the real world, I am often reminded forcefully that I am old and may not quite belong anymore. But contributing to research is an important way that I can challenge this.
- Bessie (OA 81): For years I was involved in the care of aging people within and outside of my family. I have had a front row seat to watch and assist them with their struggles as the aging process led to decreased mobility and, sometimes, decreased mental capacity. I have seen and experienced myself how difficulties in coping with aging are exacerbated by societal attitudes towards the elderly. OA-INVOLVE presented an opportunity for me to work with researchers and provide input as both a caregiver and older adult myself. My curiosity was peaked by the idea that we, as older adults, could actually be an integral part of research as co-researchers whose knowledge and lived experiences could play a significant role in developing technologies that can enhance the quality of life for older adults. It certainly was a novel idea.
- Judith (OA 78): I agree. The invitation to share with researchers my life experiences and knowledge is what drew me to participate – it appeals to my creative nature!
- Ian (OA 71): OA-INVOLVE piqued my curiosity as soon as I was introduced to it. I think all of us stepped forward because we want to contribute to the benefit of older adults’ lives. Besides, I’m fond of technology!

## Understanding roles and expectations as a research contributor

- Janet (OA 85): When I was invited to join the research team, I was not at all certain as to what my role would be. I had a hard time believing I had something to offer the process.
- Bessie (OA 81): Me too. When I was approached to become part of OA-INVOLVE, it gave me pause to consider what, if anything, I could contribute. Firstly, I was a novice with computers and technology and, secondly, my past experiences with research of any kind had always been of the more traditional variety, where people were subjects and who were not actively engaged as co-researchers.
- Janet (OA 85): I must say, despite my initial trepidation, over time I gained confidence in my abilities and insights.
- Pia (AR 54): As a researcher with OA-INVOLVE, I do believe that the perspectives and goals of older adults should be at the forefront of any social action plan aimed at bettering the world for them. We don’t just simply encourage active participation, but solicit and facilitate their participation in any activities they wish to be involved in across all phases of the research projects that we do. This way we ensure that the perspectives, interests, and identified goals of older adults are at the forefront of our research that is aimed at supporting them to live their best lives.
- Alisa (AR 41): In the theoretical literature, engagement of older adults is promoted as the best way to ensure that technologies and services meet the needs of older adults. Also known as transdisciplinary working, this is an approach to engaging older adults to uncover their tacit knowledge or lived experiences and to transcend researcher/subject boundaries. What gets less attention is that this can only happen if older adults are provided opportunities to equally engage and learn. Without the opportunity to contribute to decision-making, new technologies will not reflect their values and ideas.
- Anne (OA 79): By being involved in research I think I can at least influence those around the table rather than being out of the picture. This way I’m not out of the social stream and I still do have something to contribute. There’s a ripple effect – I genuinely don’t think anybody leaves thinking, “well that old woman doesn’t know what she’s talking about”.
- Kieran (AR 49): As an academic researcher myself, I do often wonder if we are too arrogant in presuming that we know what is best for people. Whether it’s health or mental health, aging, social problems, or any other kind of applied field. How many researchers actually bother to speak to the people they are trying to help before moving in to solve their problems? How can we presume to know what kind of help – technological or otherwise – would be most appreciated, if we don’t even have a proper conversation first?

## Challenges contributing to research

- Anne (OA 79): I find it difficult to discuss any negative experiences with research projects because I haven’t had any. Having said that, it is absolutely true that ageism is the last acceptable prejudice. Old people are often considered passive rather than active. We are expected to accept what is given to us without participating in the innovation of it, or even questioning if this product or process is what we want or need. I think it’s critical for older adults to have opportunities to spend time and share information with younger adults, especially when these younger people are researching and developing tools to assist people with the challenges they face as they get older. Anyone may be able to design a garment to assist safe mobility, but if it’s too bulky, causes loss of balance, or makes bending over a challenge, then it’s not going to be well-received.
- Janet (OA 85): A real highlight for me has been the opportunity to attend various conferences. For the most part, the people I have spoken with in these settings are welcoming of my comments, but I recall one researcher for whom the wisdom of seniors had not yet entered his thinking. He had designed a walker that would stop automatically when weight was applied to the seat, but I pointed out that many of us use walkers to carry around our things like books or groceries. I told him that this particular feature would not be useful. His response was quite dismissive.
- Bessie (OA 81): I remember one experience of testing off-the-shelf technology developed for older adults. It resulted in a ton of laughter when neither the researcher nor I could figure out how to get the thing out of the box. This proved the point to both of us that when older adults are not even consulted or involved in the development of technology designed to assist them, it is sometimes useless.
- Kieran (AR 49): Involving older adults in the research process in meaningful ways is not easy. It takes careful consideration about how best to involve people in a project, sensitivity to others’ needs and putting them before your own in many instances, and the willingness to listen with humility and accept deep down that no matter how much I’ve studied and become an expert in something, there is so much I don’t know. And all that work is worth it! It leads to better research and it leads to deeper understanding of life’s problems, beyond just surface level knowledge one might get from studying something from a distance.

## Benefits of participation in research

- Anne (OA 79): My experiences being a partner in research have all been extremely positive. These experiences have given me a feeling of still being a valued member of the community. I think these interactions have been very valuable in creating a shared understanding of each of our places in society, as well as the abilities we bring and the contributions older adults can make.
- Judith (OA 78): Being a part of OA-INVOLVE helps older adults like me to be a part of the conversation about what areas of research would be useful and relevant to pursue. This empowers us and gives value to what we are contributing. I have expanded my knowledge of health care for older adults, contributed to this knowledge, and felt valued for my ideas and contributions.
- Janet (OA 85): I got so much more from joining the OARPG than I ever could have imagined. There was one ‘photovoice’ project that required me to learn how to use an iPad – this was a totally new technology to me, but it was so rewarding to learn this new skill.
- Bessie (OA 81): I had a similar experience! Using the iPad raised some anxiety for me, because I had never used one before. The researchers worked with me to help me understand how to use it, and I ended up feeling an enormous sense of accomplishment when I was able to make a useful contribution. Being so involved in research has opened a world of technological innovation to me.
- Ian (OA 71): I too have found that gaining knowledge and sharing my experiences has been very stimulating and fulfilling. An unexpected but welcome outcome of contributing to research is that I have found the knowledge I’ve acquired helps me manage the demands of caring for my aging mother and relieving some of that stress.
- Bessie (OA 81): It has been a wonderful experience and I hope mutually beneficial for all. I believe there is a reciprocity in the relationship between us older adults and the OA-INVOLVE researchers. I hope my experiences have benefitted them in their pursuit to prove that it is beneficial for researchers to view and engage older adults as active participants in research related to aging. At the same time, I have benefitted from being valued as an older adult who can make a meaningful contribution regardless of my age.
- Ash (AR 32): Speaking from the perspective of academic researchers, the benefits we have received go beyond research outcomes and extend to the ways in which we have grown as researchers in general. What I mean by this is that our experiences collaborating with older adults has helped us grow in terms of how we work with other people – whether that’s older adults, other researchers, or members of the larger research team. The little things that traditional research teams don’t always care about, we’ve come to appreciate just how important they are to effective communication.
- Pia (AR 54): Engaging older adults as co-researchers strengthens the quality of the work that we do together. That is why it’s so imperative that older adults are treated as equal partners in research, design, and advocacy work more broadly, and that they have direct influence over the entire research, development, and implementation processes we engage in.
- Kieran (AR 49): This partnership has been fruitful and inspiring and from it we have learned so much not only about older adults’ views on particular technologies but, perhaps more importantly, about the nature of such engagements and partnerships. It also has immense potential for challenging stigma and ageist assumptions about the value and abilities of older adults in our society, and refocusing health research away from loss and decline towards strength-based empowerment.
- Ash (AR 32): Working with OA-INVOLVE and engaging older adults in technology development and research really demonstrates the impact of participatory research, and why what we do is so important. Working with older adults is like having a spotlight highlighting issues related to disability, stigma, and marginalization.
- Bessie (OA 81): OA-INVOLVE projects are great examples of cooperation, inclusion, and stellar communication between researchers and older adults. For me, personally, it has been an opportunity to gain insight into the world of research and technology, while also providing information and experience about aging and being an older adult to researchers. It has been an experience where researchers have actually listened to us.
- Ian (OA 71): It gives me hope for improved quality of life for older adults living across all settings.

## Advice for academic researchers

- Anne (OA 79): One of the issues with people innovating new products to assist older adults is that often they are addressing the needs of the caregiver or the person with the purse strings. I can understand their need to sell the product to meet their needs, but it is a difficult trade off when it means trading off the needs of the end user. I see many instances where products or programmes are being developed to ensure the physical safety of the older person in care. This is often at the expense of the person having privacy or self-determination. For myself and others my age that I know, we would certainly trade off some safety for independence and privacy.
- Bessie (OA 81): This is another example of stigma. As I have aged, I’ve often been subjected to some of the ingrained systemic and societal attitudes about older adults. This has been particularly noticeable in relation to my many contacts with the health care system. There seems to be this misconception that we all age at the same rate and in the same way, as if “the elderly” is one collective, homogeneous group and, furthermore, as we age our intelligence diminishes along with our skills, abilities, and knowledge. It is so important that researchers not harbour this misconception and instead recognize the heterogeneity of the elderly and the vast diversity in terms of our experiences and potential contributions.
- Anne (OA 79): It reminds me that the disability rights movement has a saying, “Nothing for us without us”. A similar mantra could be used for older adults. We need to have input into developments that target our demographic.

## Conclusions

While there is growing interest within the field of gerotechnology in using participatory approaches to research and design to meaningfully engage older adults in activities and decision-making, they remain rare. One notable exception is OA-INVOLVE, which actively supports older adults to participate as co-researchers in gerotechnology projects. Our comment paper presents a conversation with older adults who are active contributors to OA-INVOLVE. We identify the following key takeaway messages from this conversation:

1. *Motivations to engage in research:* Important motivations for older adults to participate in gerotechnology research include the desire to counteract societal tendencies towards ageism and ableism, and to share their lived experiences of caring for aging loved ones and their own experiences of aging in order to contribute to the enhancement of quality of life for older adults.
2. *Understanding roles and expectations:* Academic researchers must put the perspectives and goals of older adults at the forefront of the work that they do by soliciting their active and meaningful participation. Older adults may experience some uncertainty and trepidation at the outset of research involvement, but may be reassured that technical proficiency is not a prerequisite to meaningful engagement in gerotechnology research.
3. *Challenges to contribute to research:* Ageism, specifically the negative stereotype of older adults as being incapable of contributing meaningfully to research, was identified as a significant barrier to older adults’ participation. Academic researchers are themselves enmeshed in societal assumptions about aging, which can impact the ways in which older adults are engaged, or not, in gerotechnology research. It is imperative the researchers do not dismiss the views of older adults. However, it is important to acknowledge that it is not necessarily easy or straightforward to engage older adults in truly meaningful ways. It takes considerable time, effort, and sensitivity, and humility to acknowledge that despite the collective expertise of an academic research team, there are always gaps in this knowledge that the lived experiences of older adults can address.
4. *Benefits of participation in research:* A number of benefits were discussed including how meaningful engagement of older adults can lead to better research and a deeper understanding of the problems faced by this population. Individual benefits included older adults feeling like a valued member of the community and society more broadly; it is an opportunity to learn about technology, which can be stimulating and fulfilling.
5. *Advice for academic researchers:* When developing technologies for older adults, academic researchers must not harbour the misconception that this population is a homogenous group. Researchers must recognize the heterogeneity and diversity of experiences by keeping the needs of the end users in mind. Further, when developing technologies, the physical safety of the older person should not be at the cost of their independence and privacy.

The demographics of our team represent older adults who are most likely to participate in gerotechnology research, namely women who identify as White and who are well educated. As such, the experiences captured in this conversation may not reflect the experiences of older adults with other intersecting identities. Given research has already identified that intersecting dimensions of identity [19], including gender [20–22], sexual orientation [21], race [20, 21], and income [21] can pose barriers to older adults’ participation in research, PD efforts within gerontechnology should be informed by an intersectional understanding of multiple disadvantages to more fully and equitably enable participation in gerotechnology research [22, 23]. However, as our conversations suggest, the privilege that being White and educated affords does not protect older adults from ageism and ableism; these are significant barriers to participation for all older adults and will require more sustained intervention to address these within gerontechnology. It is our hope that our conversation presented here trigger the change needed to ensure that older adults are given opportunities to engage more fully in technology design and development processes.

## Data Availability

Not applicable.

## Abbreviations

OA-INVOLVE: Older Adults’ Active Involvement in Ageing & Technology Research and Development
OARPG: Older Adult Research Partner Group
